# Application of circuit simulation method for differential modeling of TIM-2 iron uptake and metabolism in mouse kidney cells

**DOI:** 10.3389/fphys.2013.00136

**Published:** 2013-06-07

**Authors:** Zhijian Xie, Scott H. Harrison, Suzy V. Torti, Frank M. Torti, Jian Han

**Affiliations:** ^1^Department of Electrical Engineering, North Carolina Agricultural and Technical State UniversityGreensboro, NC, USA; ^2^Department of Biology, North Carolina Agricultural and Technical State UniversityGreensboro, NC, USA; ^3^Department of Molecular, Microbial, and Structural Biology, University of Connecticut Health CenterFarmington, CT, USA; ^4^Department of Medicine, School of Medicine, University of Connecticut Health CenterFarmington, CT, USA

**Keywords:** circuit simulator, export, ferritin, iron, model, storage, TIM-2, uptake

## Abstract

Circuit simulation is a powerful methodology to generate differential mathematical models. Due to its highly accurate modeling capability, circuit simulation can be used to investigate interactions between the parts and processes of a cellular system. Circuit simulation has become a core technology for the field of electrical engineering, but its application in biology has not yet been fully realized. As a case study for evaluating the more advanced features of a circuit simulation tool called Advanced Design System (ADS), we collected and modeled laboratory data for iron metabolism in mouse kidney cells for a H ferritin (HFt) receptor, T cell immunoglobulin and mucin domain-2 (TIM-2). The internal controlling parameters of TIM-2 associated iron metabolism were extracted and the ratios of iron movement among cellular compartments were quantified by ADS. The differential model processed by circuit simulation demonstrated a capability to identify variables and predict outcomes that could not be readily measured by *in vitro* experiments. For example, an initial rate of uptake of iron-loaded HFt (Fe-HFt) was 2.17 pmol per million cells. TIM-2 binding probability with Fe-HFt was 16.6%. An average of 8.5 min was required for the complex of TIM-2 and Fe-HFt to form an endosome. The endosome containing HFt lasted roughly 2 h. At the end of endocytosis, about 28% HFt remained intact and the rest was degraded. Iron released from degraded HFt was in the labile iron pool (LIP) and stimulated the generation of endogenous HFt for new storage. Both experimental data and the model showed that TIM-2 was not involved in the process of iron export. The extracted internal controlling parameters successfully captured the complexity of TIM-2 pathway and the use of circuit simulation-based modeling across a wider range of cellular systems is the next step for validating the significance and utility of this method.

## Background

There have been both classical and advanced uses of analogous electronic circuit concepts in evaluating biological systems. For more than several decades, both animal and plant physiologists have used models such as Ohm's Law to model environmental response (Janes, 1970; Meier et al., 2003). A modern challenge has been to discover and interrelate cellular dynamics with higher-level outcomes (Kitano, 2002a). Biochemical systems theory (BST) provides a conceptual foundation for differential analysis of the functional requirements and design principles of a viable cell (Savageau, 1972, 1979, 2001). Electrical circuits are also subject to differential analysis of their linear and nonlinear components (McAdams and Shapiro, 1995). We propose that circuit simulation may be a powerful technique for realizing the potential of BST within the 21^st^ century discipline of computational systems biology, a field that aspires to evaluate complex biological systems through the use of computers (Kitano, 2002b).

Circuit simulation software has been extensively developed by semiconductor and electronics industries to handle circuit topologies having complex objectives for optimization and having many diverse interconnected components. Circuit simulation for biological systems was attempted several decades ago in the early years of the digital age (Thomas and Mikulecky, 1978), but usage has been infrequent. Its relevance may be renewed now both by a strong community effort to extensively crowd source the computer modeling of cells (Helikar et al., 2012), and by transformative developments such as a whole cell simulation of phenotype that illustrate the prowess of drawing together a wide range of mathematical models for cellular genome expression (Karr et al., 2012). After new experimental findings go beyond the original knowledge for the modeled system, there is a need to model the newly discovered subsystem and integrate it into the prior model. Contemporary circuit simulation software provides an agile platform for inputting a differential model and extending it with a rich feature set of advanced numerical and optimization methods.

To test a circuit simulation approach, we sought to examine iron, a cellular micronutrient for which both modeling and a new wave of experimental data exists. Outside of cells, sources of iron in the bloodstream include both transferrin (Tf) and ferritin (Ft). There are complex multicellular conditions of disease associated with increased serum levels of iron-loaded Tf and iron-loaded Ft (Konijn et al., 1981; Arad et al., 1988; Torti and Torti, 1994), and the underlying mechanisms and regulatory effects for cell-serum iron transport are complex and variable across different species (Kuchroo et al., 2003; Knickelbein et al., 2006; Rennert et al., 2006; Watanabe et al., 2007; Todorich et al., 2008; Rodriguez-Manzanet et al., 2009; Rejniak et al., 2010). Recently, a mouse-specific T cell immunoglobulin and mucin domain containing (TIM) protein receptor, T cell immunoglobulin and mucin domain-2 (TIM-2), has been found to process iron delivery. Regulatory effects of TIM-2 have been identified in both mouse brain glial cells (Watanabe et al., 2007) and kidney cells (Han et al., 2011). Han et al. (2011) found that TIM-2 uptakes iron from exogenous H ferritin (HFt). Ferritin is an iron storage and delivery protein made of both H and L subunits (Han et al., 2000; Todorich et al., 2011).

Although a mathematical model of iron metabolism in mammalian cells has been recently proposed (Chifman et al., 2012), this model does not account for the uptake of iron by TIM-2. It is based upon iron uptake through the classical transferrin-transferrin receptor pathway and storage of iron within ferritin (Klausner et al., 1983; Baynes et al., 1987). State variables account for the movement of iron between a labile iron pool (LIP) and four types of proteins—transferrin receptor 1 (TfR1), exporter ferroportin (Fpn), HFt, and active iron regulatory proteins (IRPs) (Chifman et al., 2012). In this study, we extended this model by developing governing equations for an additional set of TIM-2 dynamics and comparing outcomes of a differential model-based circuit simulation to laboratory data. Laboratory data were collected *in vitro* for different times of exposure of HFt to TIM-2 cDNA transfected mouse kidney TCMK-1 cells. The outcome of the comparison between simulation data and laboratory data showed circuit simulation to be a valuable tool for quantifying emerging knowledge of the integral and complex role that micronutrients have in cellular physiology.

## Materials and methods

### Iron uptake and iron storage data

Data and the experimental procedures for iron uptake and iron storage analysis are from (Han et al., 2011). Brief summaries of iron uptake and storage methods are described below.

#### Iron uptake study

125 μCi ^55^FeCl_3_ was added to 50 μg/ml mouse recombinant HFt in buffer of 20 μM citric acid, 2 mM ascorbate, and 0.1 M HEPES (pH 6.0) (Santambrogio et al., 1993). ^55^Fe-HFt complex was then filtered through a 0.45 μm syringe filter. Two μg/ml ^55^Fe-HFt complex was added to 1 × 10^6^ TCMK-1 vector or TIM-2 cells and the cells were incubated at 37°C for 0, 5, 15, 30, 60, 90, and 120 min. Cells were washed three times with PBS and harvested in whole cell lysis buffer [25 mM Tris pH 7.4, 1% Triton X-100, 1% sodium dodecyl sulfate (SDS), 1% sodium deoxycholate, 150 mM NaCl, 2 μg/ml aprotinin, 1 mM PMSF, complete protease inhibitor (Roche Diagnostics, Indianapolis, IN)]. The radio-activity of ^55^Fe was measured. The experiment was performed in triplicate.

#### Iron storage experiment

125 μCi ^55^Fe was loaded into dialyzed biotinylated HFt and then dialyzed in 0.1 M HEPES at 4°C. TCMK-1 TIM-2 or vector cells were incubated with 2 μg/ml biotinylated-^55^Fe-HFt at 37°C for 2 h. Plates were washed with PBS and placed in PC-1 growth media. Collection times were at 0, 2, 4, 8, 24, and 48 h after incubation. Cell lysates were prepared by homogenization in lysis buffer for 5 s, and then centrifuged at 12,000 × g at 4°C for 15 min. Biotin-^55^Fe-HFt was immunoprecipitated from the supernatant by incubation with streptavidin conjugated beads (Jackson Immuno Research, PA) (Wang et al., 2007). The beads were dissolved in 10% SDS/0.1 M NaOH solution and the ^55^Fe radio-activity was measured. The radio-activity of ^55^Fe from extracts depleted of biotinylated ferritin was also measured. The experiment was performed in triplicate.

### Chemicals and cell cultures for iron export experiment

Chemicals and cell cultures were purchased and handled as previously described (Han et al., 2011). The TCMK-1 mouse kidney epithelial cell line was obtained from the American Type Culture Collection (ATCC, Rockville, MD). Transfection of TIM-2 was performed with vector plasmids [BSR-α-FLAG (Chen et al., 2005)]. Selection for stable transfectants of TIM-2 was as previously described (Han et al., 2011).

### Iron export experiment

To test if TIM-2 can be the exporter of HFt and iron, TCMK-1 vector and TIM-2 containing cells were pre-loaded with ^55^Fe-Tf. The biotin-labeled apo-HFt was added to the cells. Biotin-HFt and ^55^Fe in media were examined as exported products.

#### Iron labeling of mouse transferrin (Tf)

125 μCi ^55^FeCl_3_ was mixed with 10 μl of 10 mM NTA (pH was adjusted to 6 using 1 M NaHCO3). The mixture was incubated with 2 mg/ml mouse apo-Tf in 0.2 M NaOAc at room temperature for 30 min. The rest of Tf binding sites were saturated with 100 μl of 10 mM cold FeNTA at room temperature for 30 min. The product was dialyzed in 0.02 M Tris-HCl (pH = 7) at 4°C overnight and the absorbance at A465 and A280 was measured (A465/A280 = 0.057, near to the ideal reading of 0.045) (Baynes et al., 1987).

#### Iron preloading and apo-HFT treatment to the cells

TCMK-1 vector and TIM-2 containing cells were incubated with 0.37 μM Tf-^55^Fe for 4 h at 37°C in culture containing 5% CO_2_. Cells were then incubated with 2 μg/ml biotin labeled apo-HFt for 3 h. Cells were washed with PBS and changed into normal growth media containing 10% FBS. Media were collected at 0, 2, 4, 24, and 48 h. ^55^Fe in media was considered exported product and measured by liquid scintillation counter.

#### Pulling down biotin-labeled HFT in the media using streptavidin beads

Media at different time points were each incubated with streptavidin conjugated beads at 4°C overnight. Beads were spun down at 4000 rpm for 5 min and then dissolved in 10% SDS/0.1 M NaOH solution and incubated at 50°C for 1 h. Activity of ^55^Fe was counted from streptavidin pulling down solution. The experiment was performed in triplicate.

### Governing equations of iron metabolism

A mathematical model of iron homeostasis developed by Chifman et al. (2012) was used as a basis for proposing an extended model with the TIM-2 pathway. Governing equations based upon this model for time derivatives (without the TIM-2 pathway) are described in Equations 1–5 with state variables *x*_1_ = [LIP], *x*_2_ = [TfR1], *x*_3_ = [Fpn], *x*_4_ = [HFt], and *x*_5_ = [Active IRPs].

(1) $${\dot{x}}_{1}=\alpha_{1}Fe_{ex}x_{2}+\gamma_{4}x_{4}-\alpha_{6}x_{1}x_{3}-\alpha_{4}x_{1}\frac{k_{54}}{k_{54}+x_{5}}$$

(2) $${\dot{x}}_{2}=\alpha_{2}\frac{x_{5}}{k_{52}+x_{5}}-\gamma_{2}x_{2}$$

(3) $${\dot{x}}_{3}=\alpha_{3}\frac{k_{53}}{k_{53}+x_{5}}-(\gamma_{3}+\gamma_{h}Hep)x_{3}$$

(4) $${\dot{x}}_{4}=\alpha_{4}x_{1}\frac{k_{54}}{k_{54}+x_{5}}-\gamma_{4}x_{4}$$

(5) $${\dot{x}}_{5}=\alpha_{5}\frac{k_{15}}{k_{15}+x_{1}}-\gamma_{5}x_{5}$$

Independent self-degrading behaviors for each state variable are captured by the terms −γ_*i*_*x*_*i*_, where γ_*i*_ are decay constants. For effects of state variable *x*_*i*_ on state variable *x*_*j*_, terms in the form of $\frac{\alpha_{j}x_{i}}{k_{ij}+x_{i}}$ represent the promoting effect, and terms in the form of $\frac{\alpha_{j}k_{ij}}{k_{ij}+x_{i}}$ represent the inhibiting effect. Activation thresholds are represented by *k*_*ij*_, and maximum production rates are represented by α_*j*_. In Equation 1, second-order reaction rates are represented by α_1_*Fe*_*ex*_*x*_2_ and −α_6_*x*_1_*x*_3_ for iron uptake and iron export, respectively. The remaining term of Equation 1, $-{\dot{x}}_{4}$, models the dynamic where, as ferritin degrades, iron is released and elevates the level of the LIP.

**Figure 1:**
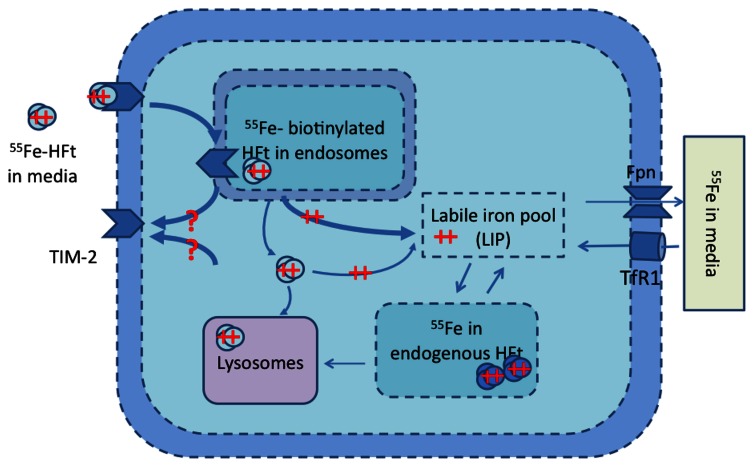
**TIM-2 pathway model of the TIM-2 receptor, endosome formation, HFt degradation, and iron release into the iron labile pool**. Iron is subsequently either stored in endogenous HFt or exported to the media. Iron uptake by the TIM-2 receptor occurs at the cellular membrane. ++ represents Fe (ferrous state), and ++ with ovals represents Fe-HFt. Unshaded ovals are for external HFt, and shaded ovals are for endogenous HFt. The question marks (?) represent pathways that do not have any direct experimental support.

The model was extended with a pathway of exogenous iron-loaded HFt (Fe-HFt) as shown in Figure 1. Four dynamic states of TIM-2 were modeled: *x*_6_ = [TIM-2 active on cell membrane], *x*_7_ = [TIM-2 bounded with HFt], *x*_8_ = [TIM-2 in endosomes], and *x*_9_ = [exogenous Ft in cell]. Time derivatives for the governing equations of this model are described in Equations 6–9.

(6) $${\dot{x}}_{6}=\gamma_{6}x_{8}-\alpha_{7}Ft_{ex}x_{6}$$

(7) $${\dot{x}}_{7}=\alpha_{7}Ft_{ex}x_{6}-\gamma_{7}x_{7}\frac{k_{47}}{k_{47}+(x_{4}+x_{9})}$$

(8) $${\dot{x}}_{8}=\gamma_{7}x_{7}\frac{k_{47}}{k_{47}+(x_{4}+x_{9})}-\gamma_{6}x_{8}$$

(9) $${\dot{x}}_{9}=\alpha_{9}\gamma_{7}x_{7}-\gamma_{9}x_{9}$$

The term α_7_*Ft*_*ex*_*x*_6_ is the combination rate of active TIM-2 with exogenous ferritin, where α_7_ is the reaction-rate constant and *Ft*_*ex*_ the concentration of exogenous ferritin. The reaction turns the active TIM-2 into a bounded one, thus this term is negative in the ${\dot{x}}_{\epsilon }$ equation, and positive in the ${\dot{x}}_{7}$ equation. The concentration of TIM-2 active on cell membrane is not only affected by the combination reaction with exogenous ferritin, but also by the process of recycling them back to cell membrane. The rate of recycling is assumed to be equal to the rate of decrease of TIM-2 in the endosome. The rate of decrease of TIM-2 is modeled by a first-order decay with a decay constant γ_6_.

The endocytosis of TIM-2 and the bounding of TIM-2 on the membrane with H-ferritin are hindered by the presence of ferritin and are expressed in Equations 7 and 8. γ_7_ is the rate for endocytosis with low concentration of ferritin (*x*_4_ + *x*_9_). The endosome formation saturation factor *K*_47_ is the threshold value of ferritin concentration when the endocytosis rate is reduced by half. Equation 9 expresses the surviving exogenous iron loaded ferritin after the endosome is dissolved and its decay rate in normal cell solution. The Equation 1 time derivative of ^55^Fe in LIP is therefore modified as shown in Equation 10. Equations 2 through 10 describe therefore the TIM-2 iron uptake and metabolism model.

(10) $$\begin{array}{l} {\dot{x}}_{1}=\alpha_{1}Fe_{ex}x_{2}+\gamma_{4}x_{4}-\alpha_{6}x_{1}x_{3}\\ \;-\alpha_{4}x_{1}\frac{k_{54}}{k_{54}+x_{5}}+\alpha_{10}\gamma_{7}x_{7}+\alpha_{11}\gamma_{9}x_{9} \end{array}$$

### Simulation

Simulation relied upon the quantified activity of ^55^Fe where concentrations from *in vitro* experiments are inferred from the strength of radiation. Assumptions were that the ratios of concentration to strength of radiation were each constant for various iron-loaded proteins. Other assumptions were that initial conditions were related to the preparation process, and that the system reaches equilibrium states before and after external experimental conditions (treatments) perturbed the system. For simplicity, this work assumed that all three iron metabolic processes (uptake, storage, and export) are in equilibrium and the level of iron concentration is below the nonlinear threshold. For this simulation, the nonlinear effects which are not related to TIM-2 are ignored. Output parameters to be compared with experimental measurements were the sum of different iron-containing components.

**Figure 2:**
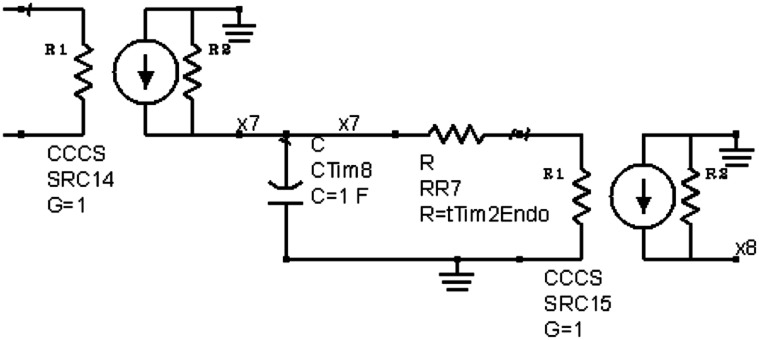
**An example circuit generated by ODE-to-circuit conversion**. The component on the left is a current-control current source (CCCS), an ideal element for current scaling. The scaling factor G = 1 for the element mirrors the current, representing iron atoms in a TIM-2/ferritin complex. The current inputs into a capacitor are to model the state of iron accumulation. At a linear release condition, the stored charge in capacitor leaks through the resistor RR7, modeling the release of iron. The current is then mirrored again on the right side of the circuit to input into another state representing iron atoms in a TIM-2/ferritin complex.

**Figure 3:**
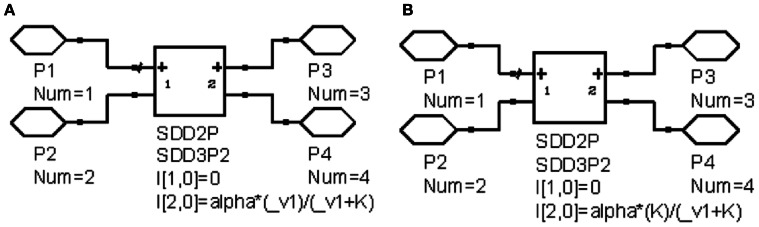
**Example circuits of nonlinear behavior. (A)** Linear to saturation model; **(B)** Constant to suppression model.

The ordinary differential equations (ODEs) for the TIM-2 pathway of iron uptake and metabolism were mapped to an equivalent electrical circuit to be implemented within a circuit simulator. The circuit simulator used in this work was Agilent Advanced Design System (Agilent ADS). Electric circuit components were mapped to the variables and equations of the TIM-2 pathway model (Figure 2). A unit capacitor was used to hold the state variables, *x*_*i*_ = *Q*_*i*_ = *V*_*i*_. Time derivative variables were represented by the current flow in and out of a capacitor, $\frac{dx_{i}}{dt}=I$. Dual directional processes were represented by a resistor connecting two capacitors, $\frac{dx_{i}}{dt}=-\frac{\left(x_{i}-x_{j}\right)}{R}$ and $\frac{dx_{j}}{dt}=\frac{\left(x_{i}-x_{j}\right)}{R}$. Unidirectional processes were represented by a current-control current source, $\frac{dx_{i}}{dt}=-\frac{x_{i}}{R_{1}}$ and, $\frac{dx_{i}}{dt}=\frac{x_{i}}{R_{2}}$. Nonlinear relations can be implemented by nonlinear-equation based sub-circuit blocks. Figure 3A shows an example of a linear to saturation model $\frac{\alpha_{j}x_{i}}{k_{ij}+x_{i}}$ term and Figure 3B shows an example of a constant to suppression model $\frac{\alpha_{j}k_{ij}}{k_{ij}+x_{i}}$ term.

**Figure 4:**
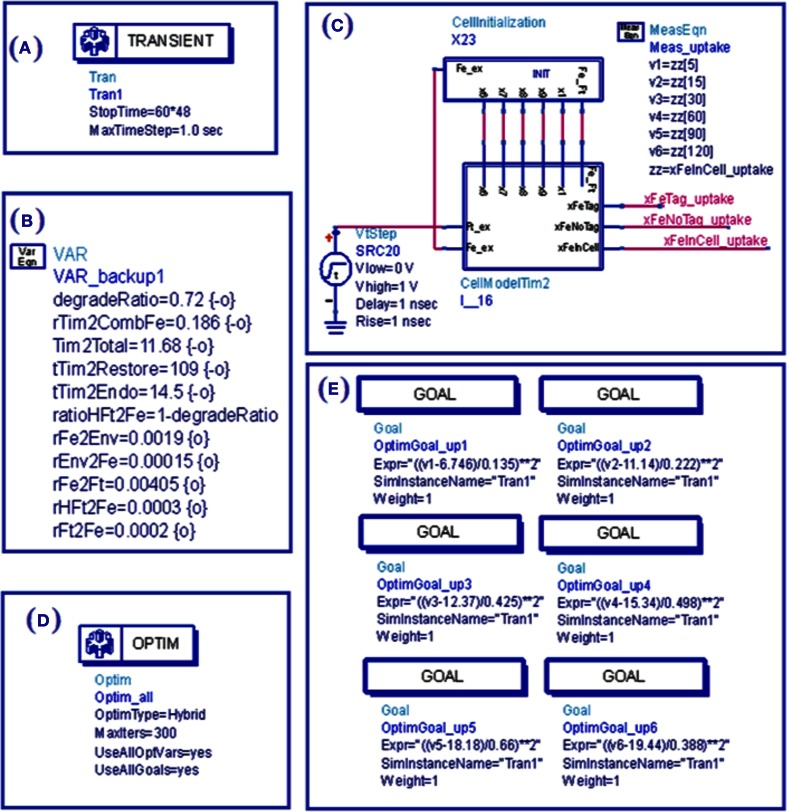
**View of ADS simulation bench for simulation of TIM-2 pathway model. (A)** Simulation setup defines the time duration and resolution; **(B)** Variable setup defines parameters and their range for optimization or tuning; **(C)** Initial condition and environment (external source) as applied to the cell subcircuit through wire connections; **(D)** Optimization setup of optimization methods and iteration time; **(E)** Goals for optimization.

A simulation “bench” was constructed with Agilent ADS as shown in Figure 4 (Agilent ADS manual, http://www.agilent.com, Santa Clara, CA, USA). With proper conversion, mathematical equations can be mapped to equivalent circuits and solved through transient simulation for dynamic process or direct current (DC) simulation for stable states. Conversion consists of three parts: mathematical equation conversion, initial condition conversion, and output parameter conversion. Controlling parameters were extracted from *in vitro* experiments of iron uptake, storage and export as were performed in TCMK-1 vector and TIM-2 containing cells. Although the experiments were performed independently, the underlying mechanisms for iron metabolism would be the same since the same cell line was used. Therefore, the model with the same internal controlling parameters should be able to describe the underlying mechanisms for these three experiments. The goal of optimization for the overall model is to find a set of internal controlling parameters that will minimize error which is modeled by the sum of squares due to normalized error (*SSNE*) as shown in Equation 11. *N* is the total number of data points collected from three *in vitro* experiments. For each point *j, y*_*j*_ represents the mean, σ_*j*_ represents the standard deviation, and *m*_*j*_ represents the modeling data. The means and standard deviations are determined from repetitions in each point.

(11) $$SSNE=\sum_{j\;=\;1}^{N}\frac{{\left(m_{j}-y_{j}\right)}^{2}}{\sigma_{j}^{2}}$$

## Results

In this study, multiple TIM-2 associated iron metabolic processes: iron uptake, storage, and export were modeled simultaneously based on a direct implementation from the Agilent ADS circuit simulator software. Internal controlling parameters for TIM-2 iron pathway were extracted by ADS based on *in vitro* data collected from mice kidney TCMK-1 TIM-2 and vector cells. The model demonstrated a capability to identify variables and predict outcomes that could not be readily measured by *in vitro* experiments.

### Iron uptake

**Figure 5:**
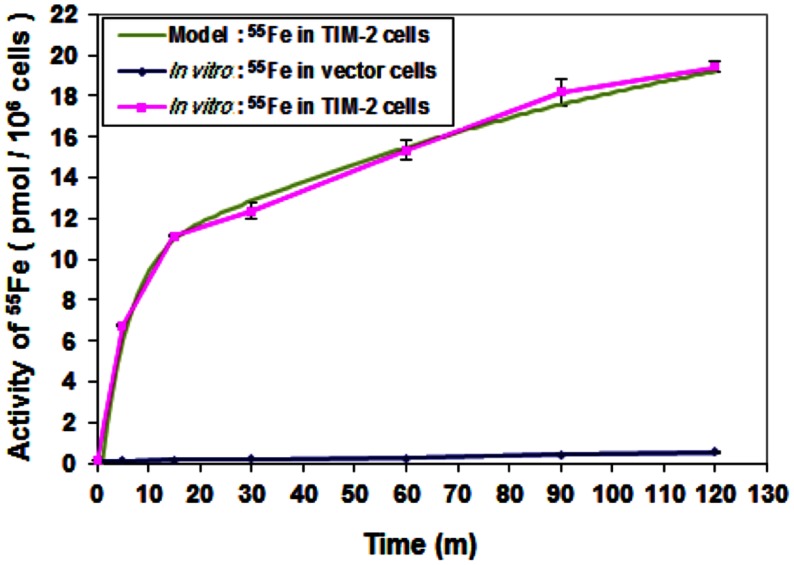
**Comparison of iron uptake rates between laboratory data and simulation**. Cells were harvested at different time points over a 2-h period and ^55^Fe amounts in cytosol fractions were counted. Data shown are means and standard deviations for triplicate replication of the experiment. The experiment was performed in triplicate.

Circuit simulation of iron uptake accurately modeled an increase in a time-dependent manner in TIM-2 transfectants, but not for vector controls (Figure 5). While the optimization of internal controlling parameters is conducted in conjunction with other experiments, the primary parameters most affected by the data of this experiment were the concentration of ^55^Fe-loaded ferritin in the cell culture (*Ft*_*ex*_), the combination coefficient (α_7_), endosome forming rate (γ_7_), and TIM-2 recovering rate (γ_6_). The iron uptake rate starts at 1.4 pmol per min per million cells, then reduces to about 0.07 pmol per min per million cells. The initial rising (within 10 min) of iron concentration is due to the combination of TIM-2 in cell membranes with Fe-HFt. The turning point at around 10 min indicates the saturation of the TIM-2 combining process, i.e., the number of available TIM-2 is largely reduced. The uptake curve is not flattened out; instead it keeps rising at a slower rate. This indicates the appearance of a new TIM-2 unit on the cell membranes, which may occur from either TIM-2 recycling or new synthesis.

### Iron storage

Circuit simulation of iron storage was consistent with iron storage occurring by the release of iron from exogenous (biotinylated) ferritin to a cellular fraction. Release of iron occurred concomitantly with degradation of biotinylated ferritin, consistent with processing through the lysosome, and in parallel with an increase in endogenous ferritin (Han et al., 2011). This dynamic and the kinetics of increase in endogenous ferritin were similar to those of degradation of ferritin in the lysosome (Radisky and Kaplan, 1998) and an empirical model established in (Han et al., 2011).

**Figure 6:**
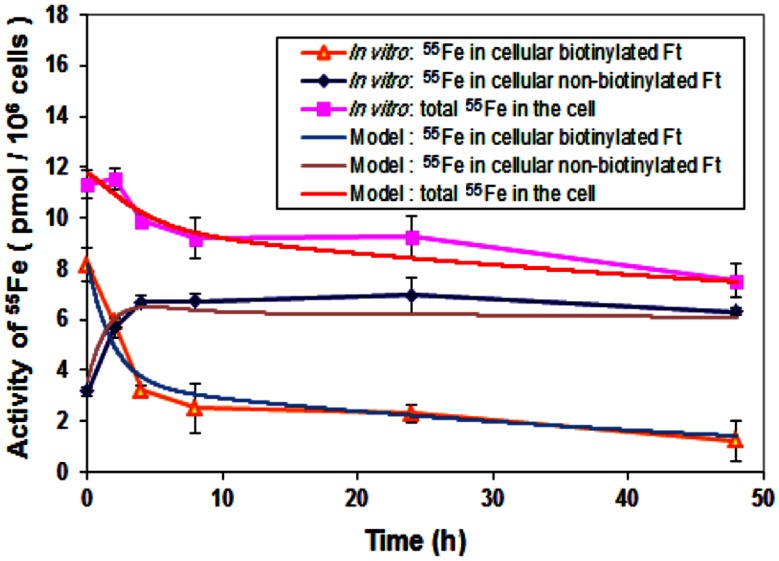
**Comparison of iron storage rates between laboratory data and simulation**. Cells were treated with biotinylated HFt loaded with ^55^Fe for various time points and the ^55^Fe amounts in biotinylated and non-biotinylated fractions were counted. Data shown are means and standard deviations for triplicate replication of the experiment. The experiment was performed in triplicate.

Degradation of biotinylated ferritin consisted of two phases with different degradation time constants (Figure 6). Phase one was for the first 4 h and phase two was for the remaining experimental period. In phases one and two, the concentration of biotinylated ferritin decreased with rates of about 20% per h and 1–2% per h, respectively. Possibly because biotinylated ferritin stayed within endosomes for approximately 2 h within phase one, it decreased faster due to the lower pH of endosome, which facilitates the ferritin degradation process in phase one. This indicates that not all of the exogenous ferritin degrades at the end of endosome (within the 2 h period) and the remaining ferritin continues degrading at a slower rate in the intracellular environment.

### Iron export

**Figure 7:**
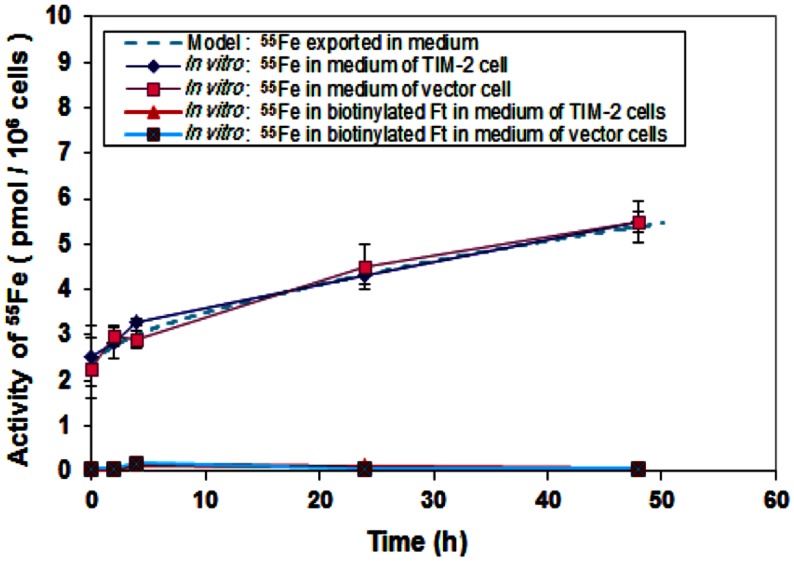
**Comparison of iron export rates between laboratory data and simulation**. Cells that had been preloaded with Tf-^55^Fe were washed and changed into normal growth media. Media were collected at different time points over a 48 h period and ^55^Fe amounts in media were counted. Data shown are means and standard deviations for triplicate replication of the experiment. The experiment was performed in triplicate.

An iron export experiment was performed for the purpose of data collection and subsequent modeling. ^55^Fe was loaded to TCMK-1 vector and TIM-2 containing cells through Tf. Biotin labeled apo-HFt was treated to the cells after iron loading. ^55^Fe and biotin labeled HFt in fresh media were measured at different time points as the exported products. The hypothesis for this study was that intracellular iron could be loaded into apo-HFt and then exported through TIM-2 receptor to the media. Our data showed that there was a general absence of biotinylated HFt in media for TCMK-1 vector and TIM-2 containing cells, and essentially all of ^55^Fe was found in total media. Figure 7 showed no difference between the amount of ^55^Fe measured in total media of TIM-2 and vector cells. This indicates that TIM-2 does not play a major role in iron export, so there is not a need for a separate export model for TIM-2 cells. For modeling, the iron export experiments would not be expected to have an effect on determining internal controlling parameters since TIM-2 was not involved in export process.

In order to confirm that the model with extracted parameters can successfully predict the outcome of the iron export experiments, simulation runs with known initial iron concentrations in the media and the cells were conducted. With the initial iron concentration of 2.4 pmol per million cells in the media and 10.8 pmol per million cells in the cell, the model predicted the experimental results within one standard deviation, starting with initial conditions within the experimental uncertainty. The additional information extracted from the model is that, for the iron present in the cells, 80% were stored in ferritin and 20% is in LIP, and the export rate of ^55^Fe from LIP is 0.2% per min for both TIM-2 and vector cells.

### Extracted parameters

The extracted parameters from TIM-2 models are listed in Table 1, and provide a reference set of values for TIM-2 iron metabolism pathway kinetics where partial iron is released from exogenous ferritin and stored in endogenous ferritin. The iron uptake values were consistent with the reappearance of TIM-2 at the cell surface to maintain an iron uptake flow. The extracted parameter, γ_6_ = 0.0142 per min, indicates the rate of recovery of TIM-2 after one uptake cycle. From model simulation, the recycling rate matched the consequence of the end of the fast degradation (in endosome) of the biotinylated HFt. This indicates that TIM-2 is recycled back to cell membrane. The rate of ferritin degradation was based on ^55^Fe measurement and detection of biotinylated HFt. The two phases of ferritin degradation indicate that amounts of iron released from the ferritin molecules were different between the beginning and the end of the 48 h experimental period. Excess iron may have been exported through TIM-2 independent pathway, which is predictably through Fpn, a known iron exporter.

**Table 1:** **Extracted controlling parameters of TIM-2 pathway model**.

**Extracted controlling parameter**	**Expression**	**Value**
TIM-2 and HFt combination rate	α_7_*Ft*_*ex*_	0.166 per min
Initial TIM-2 endosome formation rate	γ_7_	0.118 per min
TIM-2 endosome degradation rate	γ_6_	0.0142 per min
Remaining fraction of HFt at the end of endocytosis	α_9_	0.283 (unitless)
HFt degradation rate in cell	γ_9_	0.00031 per min
Saturation factor of endosome formation	*K*_47_	0.11 pmol per million cells
Direct iron uptake rate	α_1_*Fe*_*ex*_	0.00015 per min
Iron export rate through ferroportin	α_4_	0.0019 per min

## Discussion

Differential modeling with circuit simulation evaluated the kinetic processes of iron relocation across three phases: iron uptake, iron storage, and iron export. Each process was represented by governing equations. The model calculated the ratios of iron movement among different cellular compartments and confirmed the specific uptake of iron via HFt through the TIM-2 receptor. By using concentration values measured *in vitro* and applying the method of circuit simulation, precisely quantified outcomes were predicted. Specifically, controlling parameters, the ratios of uptake, storage, and export, and the recycling rate of the TIM-2 receptor were predicted and consistent with the dynamic role of the TIM-2 receptor based on iron kinetics. The continuous distribution of outcomes predicted by the simulation will ultimately, when applied to multiple host/cell lineages and environment controls, allow for focused comparisons that evaluate differences between species and physiologic conditions that impact pathways of cellular iron metabolism.

The circuit simulator simulated multiple biological processes: iron uptake, storage, and export simultaneously based on a direct implementation from the Agilent ADS circuit simulator software interface. Although the underlying processing of differential models by a circuit simulator makes itself comparable to a general performance capability of Matlab (another common tool for modeling biological systems), the Agilent ADS circuit simulator interface avoids a complicated coding process. When compared to other circuit simulators such as PSPICE, Agilent ADS provided an intuitive interface and wide range of features for multiple variable optimizations that we used for kinetic modeling. Finally, unlike software for generic mathematical modeling, circuit simulators are advantageous in having a software architecture that is scalable for a large number of ODEs, a feature that may be promising for expansive studies of reaction networks. A summary of these comparisons is provided in Table 2. An ongoing challenge in biological studies has been the resolution of analytical bottlenecks that frequently occur after the initial quantification of data. Provided that an analyst has a working understanding of linear control systems, the use of circuit simulation software when applied to kinetic modeling would be both flexible and straightforward based on the results of this case study.

**Table 2:** **Comparison between circuit simulator with Advance Design System and Matlab**.

	**Circuit simulation with Advance Design System**	**Matlab**
Solving ODE	Yes	Yes
Solving ODE system	Yes	Yes
Converging control	Select different solver both automatically and manually	Select different solver manually
Optimization	Implemented directly through interface	Implemented through programming
Multiple goal optimization	Implemented directly through interface	Implemented through programming

ODE, ordinary differential equations.

Although the usage of biotechnology to generate data is accelerating, the inexact measurement of parameters in biological systems can limit the number of ODEs that would require a high-throughput analytical capacity. We expect however that as the scope of analysis moves beyond a limited set of model organisms and biochemical pathways, the circuit simulation approach would benefit multi-level analyses that go from molecules to entire ecosystems in an evolutionary context. The degree to which the high-throughput capacity and multiple goal optimization interface of circuit simulation software are needed would depend greatly on what is found upon investigating for pluralism in nature's “circuitry.” In this study, the identification and analysis of a novel non-human pathway for a well-studied micronutrient such as iron represents a first step in uncovering such pluralism.

The simulation setup in this study was configured for a co-simulation of three experimental data sets, but the setup readily supports multiple combinations of experimental data sets to be evaluated separately or together. Prospective testing of this method for circuit simulation would include functional validation of model-based predictions. For instance, the significance of the extracted parameters in this study may be further pursued by comparison with other animal models for expressional variation with, or lack of, the TIM-2 receptor and conducting likelihood analysis for internal controlling parameters. We further anticipate that the merging together of multiple differential models would be useful for evaluating the complex, dynamic outcomes of a more extensive and biologically responsive cellular network.

The scalability and analytical feature set of circuit simulation is enormous, and compares favorably to other approaches found in computational modeling studies (Ulrich et al., 2006; Laubenbacher et al., 2009; Rejniak and McCawley, 2010; Rejniak et al., 2010, 2012; Salgado et al., 2010; Wu et al., 2010; Zhu et al., 2011). Advanced numerical methods include Jacobian matrix evaluation, step control, and convergence control, to handle huge amounts of linear and nonlinear electrical components. Optimization methods include genetic, random, gradient, minmax, random minmax, quasi-Newton, and least path. Different optimization methods can cover different situations such as the quality of initial guess, converge speed, and existence of local optimum conditions. This presents a powerful range of options for developing and extending models that are based upon ongoing research to identify new mechanisms within complex cellular networks.

A distinctive issue can often be whether the strength of a software application is to enable detailed customization of an analysis through scripting in a programming language, or to accelerate throughput and tractable project completion by pre-configured interface options. For the former approach, a general ideal is to enable a wide variety of approaches to connect data to analytical computations. For the latter approach, the variety of data-to-computation modalities can be restrictive if the pre-configured interface has incorrect assumptions and/or does not provide a wide enough range of options to manage different contexts. It is therefore an interesting finding that the interface-based ADS tool, designed for an electrical engineering context, was effective at simulating biological system parameters. The unique feature of ADS, as well as other circuit simulators, is their history of solving linear and nonlinear ODE for complex and potentially massive circuits. This case report indicates that a complex analysis may be achieved in an interface-based circuit simulator without extensive knowledge of a programming language. This capability has potential application for future complex modeling of biological systems.

## Author's contribution

Zhijian Xie and Jian Han designed the modeling study. Suzy V. Torti and Frank M. Torti designed the *in vitro* experiments and coordinated the use of experimental materials. Jian Han performed the *in vitro* experiments. Zhijian Xie, Scott H. Harrison, and Jian Han conducted the analysis and wrote the manuscript. All authors read and approved the final manuscript.

### Conflict of interest statement

The authors declare that the research was conducted in the absence of any commercial or financial relationships that could be construed as a potential conflict of interest.

## Abbreviations

ADS: Advanced Design System
DC: direct current
FeNTA: ferrous nitrotriacetic acid
Fpn: ferroportin
LIP: labile iron pool
HFt: H ferritin
ODE: ordinary differential equations
PBS: phosphate buffered saline
SDS: sodium dodecyl sulphate
SSNE: sum of squares due to normalized error
TIM-2: T cell immunoglobulin and mucin domain-2
Tf: transferrin
TfR 1: Transferrin receptor 1.
